# Supplementary material for: Evaluation of the community-based outpatient therapeutic feeding program implementation for managing children with severe acute malnutrition in Northwest Ethiopia: A mixed-method evaluation protocol
Source: PLoS One. 2022 Oct 11;17(10):e0275964. doi: 10.1371/journal.pone.0275964 (PMC9553038; doi:10.1371/journal.pone.0275964)
Supplement: S1 Checklist — (DOCX) [file pone.0275964.s001.docx]

**Questionnaire and checklist**

**Part I: Availability dimension checklists**

1. Number of trained HEWs on OTP_________________

2. The number of trained health workers working at the OTP site during data collection period? _____________

| *s.*  *No.* | ***Supplies*** | | ***Availability (Y or N)*** | |  | ***Remark*** | | | | | |
| --- | --- | --- | --- | --- | --- | --- | --- | --- | --- | --- | --- |
|  |  |  |  |  | ***Quantity***  ***(sufficient or insufficient)*** | ***appropriately stored***  ***(yes or no)*** | ***stock out***  ***(For how long)*** | | ***If stock out, write the time*** | | |
|  |  |  | **currently** | **last 6 months** |  |  |  |  |  | |  |
| 101. | RUTF | |  |  |  |  |  | |  | |  |
| 102. | Vitamin A capsule | |  |  |  |  |  | |  | |  |
| 103. | Amoxicillin tablets | |  |  |  |  |  | |  | |  |
| 104. | Amoxicillin syrup (125 mg/5 ml) | |  |  |  |  |  | |  | |  |
| 105. | Folic Acid tablets | |  |  |  |  |  | |  | |  |
| 106. | Mebendazole or Albendazole | |  |  |  |  |  | |  | |  |
| 107. | ReSoMal | |  |  |  |  |  | |  | |  |
| 108. | Standard ORS | |  |  |  |  |  | |  | |  |
| 109. | Soap for hand-washing | |  |  |  |  |  | |  | |  |
| 110. | Safe drinking water (at least one Jerry can) | |  |  |  |  |  | |  | |  |
| **s.no** | | **Materials** | | | | | | **Yes/No** | | **Remark** | |
| 111. | | SAM Protocol (2019) | | | | | |  | |  | |
| 112. | | OTP quick reference (is it in the appropriate local language?) | | | | | |  | |  | |
| 113. | | SAM classification Algorithm | | | | | |  | |  | |
| 114. | | MUAC classification table | | | | | |  | |  | |
| 115. | | ICCM protocol | | | | | |  | |  | |
| 116. | | MUAC tape | | | | | |  | |  | |
| 117. | | Functioning weight scale | | | | | |  | |  | |
| 118. | | Length board | | | | | |  | |  | |
| 119. | | Standing meter | | | | | |  | |  | |
| 120. | | Wt for Ht Reference card (WHO) | | | | | |  | |  | |
| 121. | | RUTF ration reference card | | | | | |  | |  | |
| 122. | | OTP card | | | | | |  | |  | |
| 123. | | OTP Monthly Statistics Report form | | | | | |  | |  | |
| 124. | | Referral form | | | | | |  | |  | |
| 125. | | Immunization chart | | | | | |  | |  | |

**Part II: Compliance/adherence dimension checklist**

Adapted from evaluation of community management of acute malnutrition (CMAM) Ethiopia.

| **s. No.** | **Patient-provider interaction observation checklist** | | **1–Yes 2– No**  **3 – Not applicable** | | | **Remark** |  |
| --- | --- | --- | --- | --- | --- | --- | --- |
| 201. | Bilateral pitting edema is measured | |  | | |  |  |
| 202. | Mid-upper arm circumference (MUAC) is measured | |  | | |  |  |
| 203. | Weight of a child is measured | |  | | |  |  |
| 204. | Height/length of a child measured | |  | | |  |  |
| 205. | Medical complication is checked | |  | | |  |  |
| 206. | Admission is according to correct criteria | |  | | |  |  |
| 207. | Child’s appetite is tested upon admission and during OTP follow-up sessions | |  | | |  |  |
| 208. | The child is classified appropriately | |  | | |  |  |
| s.  No. | | **Document extraction checklist** | | **1–Yes 2– Yes, but needs improvement**  **3– No,**  **4 – Not applicable** | **Remark** | | |
| 209. | | History and physical examination are recorded accurately on the OTP Chart | |  |  | | |
| 210. | | Date of admission and discharge recorded | |  |  | | |
| 211. | | Routine medication given is recorded accurately | |  |  | | |
| 212. | | Amount of RUTF needed is correctly calculated | |  |  | | |
| 213. | | OTP registration book is completed correctly | |  |  | | |
| 214. | | Beneficiaries are discharged according to the protocol | |  |  | | |
| 215. | | Correct number of absentees/defaults/ are identified for follow-up at home visits | |  |  | | |
| 216. | | RUTF is dispensed by the health workers working at OTP | |  |  | | |
| 217. | | OTP monthly statistical report is prepared correctly (death, defaulter, recovered, ) | |  |  | | |
| 218. | | OTP monthly report is sent to the next level | |  |  | | |

**Part III: Acceptability of the program services by the mothers/caregivers**

|  | **Questions** | **Responses** |
| --- | --- | --- |
| 301. | Have you ever experienced child malnutrition previously? | 1. Yes 2. No |
| 302. | Is there any transportation access to the health post? | - - - 1. Yes       2. No |
| 303. | If yes, how much it costs? | ____________birr |
| 304. | How many hours it takes (on foot)? | ____________ |
| 305. | There is access of transportation to the health post. | - - - 1. 1. Yes       2. 2. No |
| 306. | The schedule or working hours of OTP site is suitable. | - - - 1. 1. Strongly agree       2. 2. Agree       3. Neutral       4. Disagree       5. Strongly disagree |
| 306. | The HEW gives me correct instruction on how to feed the RUTF for my child (both) | Strongly agree  Agree  Neutral  Disagree  Strongly disagree |
| 307. | The HEW gives me correct instruction on how to prepare and offer complimentary feeding for my child (both) | 1. Strongly agree 2. Agree 3. Neutral 4. Disagree 5. Strongly disagree |
| 508. | The HEW appoints me when to return for the next visit. | 1.Yes  2. No |
| 309. | The amount of time spent for the services is reasonable. | 1. Strongly agree  2. Agree  3. Neutral  4. Disagree  5. Strongly disagree |
| 310. | The HEW well educates me about child malnutrition (both) | 1. Strongly agree 2. Agree 3. Neutral 4. Disagree 5. Strongly disagree |
| 311. | The HEW approach was friendly for me. | 1. Strongly agree  2. Agree  3. Neutral  4. Disagree  5. Strongly disagree |
| 312 | I am comfortable to offer RUTF for my child(both) | 1. Strongly agree  2. Agree  3. Neutral  4. Disagree  5.Strongly disagree |
| 313. | If the answer for Q13 is strongly disagree /disagree/, what is the problem? | 1. Color of RUTF 2. Taste of RUTF 3. Consistency of RUTF 4. Vomiting or diarrhea after consuming RUTF |
| 514. | Sharing the RUTF with other family members is recommended(both) | 1. Strongly agree  2. Agree  3. Neutral  3. Disagree  4.Strongly disagree |
| 315. | Selling or buying RUTF from market is possible (quali only) | 1. Strongly agree  2.Agree  3. Neutral  4.Disagree  5.Strongly disagree |

**Part IV: Socio-demographic characteristics**

|  | 1. **Socio-demographic characteristics** | |
| --- | --- | --- |
| 401. | Sex of the child | 1. Male 2. Female |
| 402. | Age of the child in months | --------------------- |
| 403. | Immunization status | - - - 1. Completed       2. Up –to-date       3. Defaulter       4. Not vaccinated       5. Unknown |
| 404. | Comorbidities | ____________(Specify) |

**Part V: Treatment outcome related characteristics**

| **Serial No** | **Premises** | **Responses** |
| --- | --- | --- |
|  | Child status after treatment | - - - 1. Recovered/cured       2. Died       3. Defaulter       4. Failed to respond       5. Transferred out |
|  | Date of admission | ____________ |
|  | Criteria for admission | 1. WFH  2.MUAC  3.Edema  4.Failure to gain weight  5. Weight loss |
|  | Date of discharge | ___________ |
|  | Age at admission | _____________months |
|  | Weight at admission | _____________Kg |
|  | MUAC at admission | ____________cm |
|  | MUAC at discharge | ___________cm |
|  | Edema at admission | 1.yes  2. no |
|  | Edema at discharge | 1.yes  2.no |
|  | Weight at discharge | _____________Kg |
|  | Length/height at admission | _____________cm |
| 10. | Length/height at discharge | ____________cm |
| 11. | Breastfeeding status | - - - 1. 1. On breastfeeding       2. 2. Non-breastfeeding |
| 11. | Vit.A supplementation | - - - 1. Yes       2. No |
| 12. | Folic acid supplementation | Yes  No |
| 13. | Types of SAM | 1. Marasmus 2. Kwashiorkor 3. Marasmic- Kwash |
| 14. | Antibiotics given? | 1. Yes 2. No |
| 15. | If “YES” for Q-10, specify | _____________________ |

**Questions to assess the knowledge of HEW**

| **So. no** | **Questions** | **Responses** |
| --- | --- | --- |
|  | Of the following select out the admission criteria to OTP **(select all that apply)** | 1. MUAC<11cm (>6months) 2. MUAC<11.5cm 3. Bilateral pitting edema 4. WFL/H <-2SD 5. WHL/H <70% |
| 2. | What are the medical complications following SAM? **(select all that apply)** | 1. Anemia (severe palmar paler) 2. Convulsion/apathetic 3. Hypothermia (<340c) 4. Hypothermia (<350C) 5. Intractable vomiting 6. Hyperthermia (>390C) 7. Hyperthermia (>38.50C) 8. Persistent diarrhea 9. Lower reparatory tract illness 10. Poor appetite 11. Eye signs of Vitamin A(bitot’s spot) |
| 3. | For whom appetite test should not be done? | 1. Children aged between 6-12 months 2. Children younger than six months 3. Children aged 6-23 months 4. Children older than 6 months |
| 4. | When do we say a child passed for appetite test? | 1. If a child consumes some part of the RUTF within 30 minutes 2. If a child consumes some part of the RUTF between 30 and 60 minutes 3. If a child consumes some part of the RUTF between15 and 30 minutes |
|  | What would you advise a mother/caregiver to examine the appetite test? **(select all that apply)** | 1. Gently offer the RUTF 2. Give the RUTF in quit and a separate area 3. Instruct the mother to wash her hands 4. Give more water to the child 5. Explain the procedure to the caretaker 6. Encourage the child to eat forcefully |
|  | When do you say a child has complicated SAM? **(select all that apply)** | 1. SAM plus severe anemia  2. SAM plus pneumonia  3. SAM plus heart failure  4. SAM plus tonsillitis  5. SAM plus poor appetite  6. SAM plus acute diarrhea  7. SAM plus severe dehydration  8. SAM plus convulsion |
|  | Where children with complicated SAM should be treated? | 1. Stabilization center in inpatient 2. OTP |
|  | For whom home visit should be done? **(select all that apply)** | 1. Are losing weight or failure to gain weight 2. Have deteriorating medical conditions 3. Children younger than six months and had discharged from SC 4. Have refused referral to SC 5. Have failed to respond the treatment 6. Are absent or defaulted the treatment 7. Bilateral edema not reduced by the third week of the treatment 8. A child consumes <75% of the RUTF of a week by third visit |
|  | Amoxicillin should be given to all children with SAM regardless of the clinical manifestation of any illness? | 1. True 2. False |
|  | What is the dose of Amoxicillin that should be given to children with SAM? | 1. 25mg/kg BID for five days 2. 20mg/kg BID for five days 3. 50mg/kg TID for five days 4. 25mg/kg BID for seven days 5. 20mg/kg BID for seven days |
|  | What is defaulter means? | 1. Absent from the regular visit for 2 consecutive visits 2. Absent from the regular visit for greater than 3 consecutive visits 3. Absent from the regular visit for greater than 1 consecutive visit 4. Absent from the regular visit for greater than 4 consecutive visits |
|  | Relapse cases should be referred to SC? | 1. True 2. False |
|  | Which category of children need to be referred to SC? **(select all that apply)** | 1. As long as mothers/caregivers request to go to SC 2. Two consecutive weight loss (non-edematous children) 3. Three static weight gain (edematous) 4. Not responding to treatment 5. Severe palmar paler |
|  | Of the following which is/are reasons for failure to respond to the SAM treatment **(select all that apply**) | 1. Non-adherence to the prescribed antibiotics 2. Failure to adhere to the RUTF protocol 3. Poor patient’s evaluation and missed medical complications 4. In appropriate guidance 5. Failure to visit the health facility as per the appointments 6. Inadequate intake or sharing of RUTF and/or medicines |
| 15. | When do you discharge the child from OTP? | 1. When the MUAC ≥12.5cm 2. When the MUAC 11 to 12.5cm 3. When the WFH/L ≥-2SD 4. No bilateral pitting edema for 2 consecutive visits 5. When the edema is downgraded from grade III to I |
| **s.no.** | **Checklist to examine the diagnosis skill of HEWs** |  |
|  | Does the HEW examine both feet to check edema? | 1.Yes  2.No |
|  | Does the HEW check for wasting? | 1. Yes 2. No |
|  | Does the HEW measure height/length appropriately? | 1. Yes 2. No |
|  | Does the HEW assess weight gain or ineffective feeding or failure to gain weight (children aged 0-6months) | 1. Yes 2. No |
|  | Does the HEW classify appropriately based on the WHO WFH/L growth curve assuming the sex of the child? | 1. Yes 2. No |
|  | Does the HEW measure the MUAC (> 6 months)? | 1. Yes 2. No |
|  | If yes? How was the measurement (in cm)? | ___________ |
|  | Does the HEW rule out the medical complications? | 1. Yes 2. No |
|  | Does the HEW asses for RUTF appetite test (6-59 months) | 1. Yes 2. No |
|  | Does the HEW put the correct diagnosis? | 1. Yes 2. No |
|  | Does the HEW pass a correct decision on where to treat children? | 1. Yes 2. No |
|  | **Checklists to examine the management skill of HEWs** |  |
|  | Does the HEW refer a child with medical complications or failed appetite test to SC? | 1. Yes 2. No |
|  | Does the HEW order RUTF for one week as per the weight? | 1. Yes 2. No |
|  | Does the HEW order appropriate doses of antibiotics (25mg/kg BID for 5 days)? | 1. Yes 2. No |
|  | Does the HEW check for vaccines (appoint after four weeks to give measles vaccine) (**if appropriate**) | 1. Yes 2. No |
|  | Does the HEW asses and appoint to provide Albendazole and Mebendazole? (>24 months) | 1. Yes 2. No |
|  | Does the HEW appoint a child after one week? | 1. Yes 2. No |
|  | Does the HEW discharge the child based on appropriate parameter/s (MUAC>=12.5cm, or no bilateral edema, and/or WFH/L >-2SD) | 1. Yes 2. No |
|  | Does the HEW forward all recommendations to the mother/caregiver during discharging a child? | 1. Yes 2. No |

Thank you!
