# Supplementary material for: Evaluation of the community-based outpatient therapeutic feeding program implementation for managing children with severe acute malnutrition in Northwest Ethiopia: A mixed-method evaluation protocol
Source: PLoS One. 2022 Oct 11;17(10):e0275964. doi: 10.1371/journal.pone.0275964 (PMC9553038; doi:10.1371/journal.pone.0275964)
Supplement: Attachment — Submitted filename: Point-by-point response-Plos.docx [file pone.0275964.s002.docx]

**Point-by-point response**

Dear editor and reviewers, thank you for giving us critical comments. The comments have a paramount positive impact on improving the readability and acceptability of the manuscript. We have provided responses to each concern and question. Kindly find it here below.

Editor’s comments:

1. In the Methods section please provide additional information regarding the qualitative study methodology. In particular, please describe how participants will be recruited for the study, whether an interview guide will be used, and the training and background of the interviewers. Furthermore, please provide additional information regarding the questionnaire development and validation. Please also include a copy of the questionnaire as supporting information.

**Response:** Comment accepted, and necessary revisions have been made. A copy of the questionnaire has been attached. Moreover, the validity and reliability procedures are now well presented in the revised version of the manuscript.

2. We suggest you thoroughly copyedit your manuscript for language usage, spelling, and grammar. If you do not know anyone who can help you do this, you may wish to consider employing a professional, scientific editing service.

**Response:** the comment has been accepted. A native academic speaker did the proofreading.

3. In your Data Availability statement, you have not specified where the minimal data set underlying the results described in your manuscript can be found.

**Responses:** Commented accepted. Although this is a protocol that doesn’t obviously include results, we have described the data availability plan.

**Reviewer #1**

1. More detail is required on the proposed methods to ensure sufficient detail to allow replication/reproduction. For example, what approach will be taken for thematic analysis (inductive, deductive, etc.) or describe what type of thematic analysis (reflexive, codebook, etc.).

**Response:** Commented accepted.

2. An implementation evaluation framework is described but there is no reference to relevant sources that support the approach proposed. There is no mention to appropriate reporting guidelines to ensure the rigorous reporting of results.

**Response:** Dear, we have cited the source from which we have taken the frameworks. You are right; there should be reporting guidelines for each study research approach employed. We have accepted the comments. See the updated version on page…

3. While there is mention by the authors of the data being available on completion of the study it is not clear that this will be in an open-source repository?

**Response:** The data availability plan has been described. We will supply the dataset as supplementary material while submitting the manuscripts for publication.

**Reviewer #2:**

1. My primary concern is that the manuscript mentions that the study data collection completes by the 30th of December 2021, and after the completion of data collection, the publication of the protocol limits the purpose of the publication.

**Response:** Dear, we genuinely share your concern, and we believe that the issue should be raised. Nevertheless, the protocol was submitted back in August, and the review process is unfortunately delayed. Considering the nature of the manuscript (protocol), we had contacted the journal several times to expedite the reviewing process; unfortunately, it wasn’t possible. However, although now the data collection has been finalized, the actual data collection had been delayed because of the COVID and local security issues, and we believe that publishing the protocol still is worthwhile. Dear, we believe that publishing the protocol would provide knowledge to the prospective scholars to replicate and follow whenever they want to carry out similar studies; it will serve as a guide. We trust that it’s rare to see the protocols of evaluation studies published. Thus, this will be served as a guide. In fact, the full-length articles that will be produced and published from the project may serve as a guide, however, the objectives of the project will be split and published separately. Thus, comprehensive information for future researchers who want to evaluate the same program may not be available. All in all, although the study has already been completed, the protocol will help prospective scholars in planning the same study in different corners of the world.

2. The manuscript failed to document the triangulation/integration of quantitative and qualitative approaches.

**Response:** Comment accepted, and detailed descriptions of the procedures are now presented in a separate subheading.

3. Moreover, the methods section of this manuscript is vaguely presented and discussed the proposed data collection, management, role and responsibilities of its team, and analytical methodology which needs a clear explanation to understand the study and implementation. For example, more description of research design, study area, assumptions for sample size calculation, sampling framework, inclusion/exclusion criteria, analytical framework/analysis of data, timeframe, and data safety and sharing mechanisms.

**Response**: Dear, exact and relevant information has been provided. Comment accepted, and we substantially revised the section considering the untouched areas. Of the responsibilities of each team member, we believe it’s uncommon to see such narrations in such protocols, and we didn’t find this in any reporting guidelines available in the EQUATOR network (<https://www.equator-network.org/>). Of course, during a systematic review, it’s common to see in protocols who will do what. However, if you believe it is necessary, we are ready to modify it in the next revision.

Dear, we didn’t calculate the sample size, as there are few participants/cases (health posts) in the three districts. We planned to consider all health posts and HEWs in the districts. Similarly, we didn’t use any random sampling technique and didn’t present a sampling frame; we will employ participants consecutively. We could have incorporated more than three districts and used a random sampling technique; however, there are limited resources (time and money). The details are presented under ‘sample size and sampling procedure’ on page #4. Dear, we don’t have particular criteria to exclude participants other than the criterion we have already stated for exclusion criteria. The data analysis, study design, and area are precisely presented. Moreover, the ethical consideration section explains the data safety and sharing mechanisms.

4. Also, the manuscript has not followed any standard guidelines and lacks a clear definition and measurement of outcome-specific indicators and their components/ validation of tools/scales used.

**Response:** Dear, as you know, there is no unique guideline for evaluation studies to prepare and report the protocol, unlike systematic review and trial studies. However, as the approaches we will follow are qualitative and quantitative studies, we have followed guidelines developed for observational (cross-sectional) and qualitative studies Consolidated Criteria for reporting qualitative research (COREQ). The manuscripts will be reported according to the respective recommended guidelines. We have added the procedures used to frame the protocol in the newly revised manuscript.

**Sources:**

<https://cdn-links.lww.com/permalink/acadmed/a/acadmed_89_9_2014_05_22_obrien_1301196_sdc1.pdf>

<https://academic.oup.com/intqhc/article/19/6/349/1791966>

<https://www.equator-network.org/wp-content/uploads/2015/10/STROBE_checklist_v4_cross-sectional.pdf>

**Note:** *we didn’t supply those reporting guidelines as there are no separate guidelines for protocols, as noted earlier.*

5. The definition and measurement of outcome variables need further discussion. In other words, though the protocol proposes multiple composite indexes (viz., for Availability, Acceptability, Compliance, and indicators of knowledge about uncomplicated SAM) to address the study objective with different scales, the authors failed to describe the components of each of the indexes and its validation.

**Responses:** Comment accepted, and necessary modifications have been made. We have provided very brief information regarding the validation in the data quality section. We didn’t give each outcome headings not to repeat texts and make the reading tedious. As the validation and reliability assuring procedures are common to all variables, we don’t believe that a detailed description of all outcomes is required.

6. The write-up of the protocol is too clumsy. Most of the time, readability is compromised and requires structural and careful review with professional language editing. The manuscript ends rather abruptly.

**Response**: An academic native speaker has done proofreading.

Thank you!

Wubet Worku Takele(t)he corresponding author
