# Supplementary material for: Evaluation of the community-based outpatient therapeutic feeding program implementation for managing children with severe acute malnutrition in Northwest Ethiopia: A mixed-method evaluation protocol
Source: PLoS One. 2022 Oct 11;17(10):e0275964. doi: 10.1371/journal.pone.0275964 (PMC9553038; doi:10.1371/journal.pone.0275964)
Supplement: Attachment — Submitted filename: Response to Reviewers.docx [file pone.0275964.s003.docx]

**Date: 29/06/2022**

**Point-by-point response**

Dear editor and reviewers, thank you for giving us critical comments. The comments have a major positive impact on improving the readability and acceptability of the manuscript. We have responded to each concern and question. Kindly find it here below.

**Reviewer #1**

1. The context/description of the C-OTP program remains vague. I suggest the inclusion of a diagram or similar illustration to describe the care pathways.

**Response:** Dear, your comment is found to be valid. We have added detailed descriptions of how the program is being operated.

2. There is frequent reference to the 'protocol' - I am assuming this is a protocol for delivering the C-OTP. However, it could also be the SAM guide (reference 19). Could an apparent reference be provided and the protocol made available as supplementary material?

**Response:** Yes, you are correct that ‘protocol’ refers to the SAM guideline. We use the term treatment protocol/guidelines interchangeably. We have revised the manuscript. The country uses the national SAM guideline for both in-patient and outpatient SAM management. There is no unique guideline for C-OTP.

3. There is some explanation as to who is carrying out the data collection provided at the end of the methods section. More clarity as to their exact role would be appreciated. For example, lines 204-205 - what are the 'departments' that are referred to (clinical/university/government?).

**Response:** comment accepted.

4. Suggest the authors reconsider or define the use of data saturation as an indicator that all available information has been gathered using qualitative methods (lines 122).

**Response:** comment accepted. The definition of data saturation has been provided in the revised version of the manuscript.

5. The access framework proposed is from 1974 (line 125). Given the recent significant development of implementation frameworks, why have the authors chosen this?

**Response:** dear, you are correct that there are interesting frameworks to evaluate the implementation of a program, specifically the barriers and facilitators, including the Consolidated Framework for Implementation Research (CFIR) and Theoretical Domains Framework (TDF). Initially, we tried to design the tools and interview guide considering the constructs of CFIR, nevertheless, we decided that some of the constructs may not fit to our objective, and we believe that the access framework would fit our study. There is an updated framework developed by the WHO which supports the access framework (<https://www.who.int/publications/i/item/9789241502894>. Even the nomenclature of the framework and dimensions included are identical, ‘access framework’. We also decided that the qualitative part should be presented in using thematic analysis. Thank you for the wonderful insight, and we will think of applying such frameworks in our future work to better design tools/guide and report findings.

6. Line 133 - the definition of 'availability' is confined to equipment/resources required to support the C-OTP. This seems at odds with the research aim and program provision. It is also not the definition held within the cited access framework reference. A reference is required for the Hawthorne effect (line 217).

**Response:** Dear, we can see your critical view. Yes, the definition of ‘availability’ is beyond checking the physical availability and functionality of essential medical equipment in a given healthcare institution. It would mean the service/program availability in a particular healthcare institution. As you said, this is clearly described in the cited article. This concept is partly addressed by our study’s ‘acceptability’ domain. That section addressed the waiting time, travel time, and so forth. Our study defines availability as the physical availability and functionality of essential equipment. For this, we used another framework developed by the World Health Organization (WHO) (<https://www.who.int/publications/i/item/9789241502894>). Still, the concept is under access framework. We have cited the article in the revised manuscript.

As to the concern of providing reference to a strategy designed to mitigate the Hawthorne effect, we have modified the strategy that we used. It’s an interesting comment. We have been waiting for the opportunity to revise the statement based on the actual strategy we have applied during the actual data collection. Dear, the previous strategy was based on what we hear/learned from expertise and senior scholars, but we couldn’t get evidence to support it. Some scholars suggest that method (discarding recordings) if the design is ‘grounded theory’, which is not similar to our aim. We then change our minds and apply other strategies that are backed by evidence. The detail is presented in the revised version of the manuscript.

7. What framework/theory will support the deductive analysis of the qualitative data? The authors mention 'existing literature' - has an evidence synthesis of existing literature been performed? (line 234-235).

**Response:** Dear, sorry for not updating the manuscript. Initially, as described earlier, we had planned to employ a framework, namely CFIR, to gather and analyse the data. However, after reviewing the constructs of this framework, we decided not to apply it. We rather planned to use thematic analysis and now we are analysing the data using thematic analysis referencing the Braun & Clarke’s guide for analysing qualitative data using the thematic analysis approach. <https://www.tandfonline.com/doi/pdf/10.1191/1478088706qp063oa?needAccess=true>

Yes, indeed, we have reviewed some literature to prepare our interview guide in order not to miss significant attributes/concepts that need to be explored. Although we didn’t review exactly similar studies, we have reviewed studies done in healthcare aiming at exploring factors affecting the level of implementation of health services/programs. Then experts in the field have tried to conceptualize the questions to prepare a flexible and comprehensive interview guide. Some themes will be generated based on the previous studies’ themes with or without modifications. For example, one of the elements of the interview guide we adopted from the literature was ‘miss use of therapeutic foods.’ While developing our guide, including this concept would help yield relevant information on the consumer’s side. Thus, we will have a theme stating the how mothers/caregivers use the given therapeutic food.

8. What methodology does the 6-step process of qualitative analysis and coding refer to? (I'm assuming Braun & Clarke?).

**Response**: yes, we will follow Braun & Clarke’s six phases of qualitative thematic data analysis. <https://www.tandfonline.com/doi/pdf/10.1191/1478088706qp063oa?needAccess=true>. We have cited the article.

9. Line 240: I am unclear whether the "questions and concerns" to discuss at the interview arise from previously completed surveys/questionnaires? A timeline to support the flow process of the evaluation study would help to clarify.

**Response**: Dear, you are correct that the message was confusing. It seems to be a sequential mixed-method approach will be used. We have modified the statement to avoid confusion. Dear, we have collected the data (both qual and quant) parallelly if possible. In exceptional cases, the quantitative preceded the qualitative data, but it doesn’t mean the approach is sequential instead, it’s to manage the data collection.

10. This has much improved, but there are some unresolved grammatical errors. There are acronyms used throughout that are not fully explained at first use, e.g., MUAC. There is also inconsistency in the application of C-OTP (lines 6, 27 and 45). This should be double-checked throughout the manuscript. I would also suggest that appropriate language is considered in the discussion, line 273, and the use of "victims" to describe the population of interest in this research.

**Response:** comment accepted and a detailed revision has been made.

**Reviewer #2**

1. Strengthen methods section of this protocol and needs clear explanation to understand project. The protocol has not discussed role and involvement of a statistician and the data analysis plans also vaguely discussed in this protocol (instead of ‘binary Binary logistic regression analysis will be conducted’, discuss/list out the type, definition and measurement of select outcomes/dependent variables along with possible independent variables).

**Response:** the comment is found to be valid and the necessary revision has been made. A significant change has been made.

2. Discuss the data collection and management process, data entry (electornic/double data entry, data quality validation and assurance

**Response:** Comment accepted and we have incorporated it in the revised document. The data quality assurance techniques are described in the ‘data collection tools, procedures, and quality assurance’ section.

3. Once the data collection completed before the publication of the protocol, it is suggested to present the date of completion of data collection and final status of data along with any inclusions/exclusions, missing values, etc.

**Response:** Dear, your comment is appreciated. However, as you know in principle, a study protocol is supposed to be published/disseminated before the actual work begins. If we put the actual date of completion, excluded/included participants, and how the missing data was managed, we feel it seems a manuscript of an actual study and may confuse participants. Dear, all the plans regarding the above-mentioned points are already presented in the protocol.

Scholars usually recommend considering an ‘amendment plan’ during preparing a protocol and some journals recommend to incorporate this although it is not available in any of the protocol reporting guides like PRISMA-P and SPIRIT. In this sense, to increase transparency, level of flexibility, and clarity, we have added an amendment section in the revised version, which is the recommended practice while preparing a protocol. However, if you feel the issue is major and needs to be considered, we are delighted to incorporate it before the protocol is published and released online.

4. Discuss the filed level operation issues and lessons learnt; possible measures adopted etc., during the study data collection/implementation.

**Response:** Dear, we highly value your comment. We lack the confidence to incorporate the points raised here as we couldn’t find a reference to cite. It’s not associated with loss of trust. As you know, scholars are recommended to follow a scientific format to prepare and report studies. We try to review a protocol reporting guidelines; no guideline like SPRIT (for trail studies) and PRISMA-P (for systematic review and meta-analysis) that contains items stated regarding your points to be considered when preparing and disseminating a protocol once an actual study is finalized or begun. Nevertheless, if it’s your strong recommendation to incorporate this point in the protocol, we are ready to accept your comment.

**SPRIT:** <https://www.spirit-statement.org/wp-content/uploads/2013/01/SPIRIT-Checklist-download-8Jan13.pdf>

**PRISMA-**P: <https://pubmed.ncbi.nlm.nih.gov/25554246/>

**Note:** these checklists are not relevant and applicable to our study. It’s just to show the concerns you raised are not common to be considered while preparing and publishing study protocols.

Thank you!

**Wubet Worku Takele (the corresponding author)**
