# Supplementary material for: Evaluation of the community-based outpatient therapeutic feeding program implementation for managing children with severe acute malnutrition in Northwest Ethiopia: A mixed-method evaluation protocol
Source: PLoS One. 2022 Oct 11;17(10):e0275964. doi: 10.1371/journal.pone.0275964 (PMC9553038; doi:10.1371/journal.pone.0275964)
Supplement: Attachment — Submitted filename: Point-by-point response-Plos.docxR3.docx [file pone.0275964.s005.docx]

**Date: 08/09/2022**

**Point-by-point response**

Dear Editor and reviewers, thank you for giving us critical comments. The comments have a significant positive impact on improving the readability and acceptability of the manuscript. We have responded to each concern and question. An academic native speaker has edited the language.

Kindly find it here below.

**Reviewer #1**

1. Thank you to the authors for their careful attention and further development of this manuscript. I have some minor issues with the manuscript that I outline in the uploaded word document that details suggested amendments to the text.

**Response:** dear reviewer, we are grateful for the comments. We have fully incorporated the editorial and typo comments.

2. Line 133-136: It is unclear if the focus group discussions will contribute to the qualitative data. Consider the position of the sentence about the logic of this section describing procedures.

**Response:** comment has been accepted, and the sentence has been re-arranged.

3. Line 154, p6: clarification required that ‘good acceptance’ is defined as mothers answering to a consensus of “agree/strongly agree” rather than the total number of mothers responding to the questions (where any answer might be given).
4. **Response:** Dear, if we get your concern, the definition needs to be based on the mother’s response, not the proportion of mothers/caregivers who have ‘agreed/ disagreed’. We have revised the statement to clarify more.
5. Table 1: remove the gender pronouns from row 5 describing compliance/adherence measures.

**Response:** your comment has been accepted.

6. Line 235: Please clarify if it is a nutritionist or a statistician who will perform the data analys.s? I assume that the nutritionist will be ably assisted by a statistician in analysing and interpreting the data based on the previously described methodology.

**Response:** Yes, you are correct that the nutritionist will do the analysis; we assume the nutritionist is knowledgeable about the variables and their definition; the nutritionist will do the analysis and be supported by a statistician. We have revised the document.

7. Line 265-266: Can you define what is meant by “level the generated themes” – is it refining, defining and naming the themes?

**Response:** Dear, sorry for the typo error. It was to say ‘label/name’, not level.

8. Line 11: suggest the use of the word ‘factors’ instead of “indicators” to describe those elements of the programme that can be evaluated for implementation and avoid the repetition in line 12.

.

**Response:** Dear, we appreciate your perspective. Thank you for giving this comment. However, as can be recalled from our previous response, ‘indicator’ is an appropriate technical term used to describe the detail of programs. The evaluation is performed based on this key indicator. When we say ‘indicator’, we are not referring to factors(barriers/enablers) contributing to the program’s implementation. All in all, we prefer to use the term indicator over factor. Following your comment, we authors have discussed and have sought an explanation from one of the evaluation experts (co-author), and we have agreed to continue with ‘indicator’.

**Reviewer #2**

1. While the authors have made some amendments to the manuscript which improve the text, the concerns I have in the analysis of data /results, remain and failed to address the concerns raised in the first round of the review process on integration/triangulation of results from both qualitative and quantitative approaches (mixed-methods), adequately. The authors suggested discussing the rationale for using both qualitative and quantitative approaches and how they combine the elements of both qualitative and quantitative research approaches to address the study objectives. Since my primary concern is that the manuscript failed in triangulation/integration of results. Moreover, the manuscript contains insufficient data and explanations in addressing the crucial aspects of the study.

**Response:** Dear reviewer, thank you for the comment. We believe we have provided rich information on how the qualitative and quantitative data will be integrated (see page 12). Dear, as you know, this is a protocol (it doesn’t include results), and we don’t have the data yet to show the actual integration. What we have discussed in this section is the plan.

2. Among the minor concerns about the methodology, the authors suggested to discuss on the study setting, data collection process, selection and recruitment of sample participants etc., in order to avoid data dredging. Moreover, I feel the manuscript is certainly less disjointed (study implementation, linkage with the qualitative results, utilization of results from both qualitative and quantitative data analysis) and I would urge the authors to have another good 'go' at the report, post-acceptance, as I am sure they will be able to make further minor improvements in structure and presentation. Also avoid typo errors (for instance, “Similarl,”; “Ethiopi a” etc.). I would also suggest someone who has statistical knowledge and, just to knock off the rough edges on the definition and measurement of the dependent variables like knowledge of HEWs about SAM; compliance of HEWs to the C-OTP protocol; acceptability of C-OTP of the service; severely malnourished children etc.

**Response:** we have incorporated the editorial comments. For your information, we have proposed the measurement strategies after consulting a statistician. We will update the analysis if we find a revision is required after discussing it with co-authors, including the statistician.

Thank you!

Wubet Worku Takele (the corresponding author).
