# Supplementary material for: Evaluation of the community-based outpatient therapeutic feeding program implementation for managing children with severe acute malnutrition in Northwest Ethiopia: A mixed-method evaluation protocol
Source: PLoS One. 2022 Oct 11;17(10):e0275964. doi: 10.1371/journal.pone.0275964 (PMC9553038; doi:10.1371/journal.pone.0275964)
Supplement: Attachment — Submitted filename: PONE-D-21-25442_R2.pdf - Reviewer comments.docx [file pone.0275964.s004.docx]

This protocol aims to report the procedure and methods to evaluate a community-based programme delivering timely and therapeutic care to children who experience severe acute malnutrition. The authors seek to describe the barriers and enablers that impact on the implantation of a programme that integrates levels of health care and relies on the competence of health education workers working with families. Therefore, the protocol is required to describe how the complexity of programme implementation can be explored rigorously and contributes to open science. A strength of this paper is the authors use of mixed-methods towards a comprehensive and holistic exploration of the factors of programme implementation.

Thank you to the authors for their careful attention and further development of this manuscript. I have some minor issues with the manuscript that I now outline.

Line 133-136: It is not clear if the focus group discussions will contribute to the qualitative data. Consider the position of the sentence in relation to the logic of this section describing procedures.

Line 154, p6: clarification required that ‘good acceptance’ is defined as mothers answering to a consensus of “agree/strongly agree” rather than the total number of mother responding to the questions (where any answer might be given)

Table 1: remove the gender pronouns from row 5 describing compliance/adherence measures.

Line 235: Please clarify if it is a nutritionist or a statistician who will perform the data analysis? I assume that the nutritionist will be ably assisted by a statistician to analyse and interpret the data based on the previous described methodology.

Line 265-266: Can you define what is meant by “level the generated themes” – is it refining, defining and naming the themes?

I also have some suggestions in relation to grammar and typos throughout the manuscript:

Line 1: Malnutrition results in 3.1 million global child deaths annually, accounting for 45% of all mortality among under-fives’.

Line 5: insert full wording with C-OTP in brackets

Line 7: suggest the insertion of the word ‘population’ before “coverage and access”, if this is what is meant by the authors.

Line 11: suggest the use of the word ‘factors’ instead of “indicators” to describe those elements of the programme that can be evaluated for implementation and avoid the repetition in line 12

Line 11-12: correct grammar “The program has multiple factors from input ~~through the impact~~ to outcomes that could be evaluated using various evaluation dimensions and model indicators.”

Line 13 suggest rewording “three-fourths” to “three quarters of…”; typo “majorities” should read “majority”

Line 14: suggest removal of the word “considerably”

Lines 13-16: a very long sentence. Consider breaking it up into 2-3 sentences.

Line 17: consider rewording to “The traditional in-patient care model of a therapeutic feeding center (TFC)…”

Line 22-24: Suggest reword to “It is therefore vital to deliver C-OTP ~~presumed~~ that is integrated with a facility-based approach and implemented on a large scale with competent HEWs to mitigate the death of hundreds of thousands of children.”

Line 32: suggest “likewise” is replaced with “however”

Line 36: suggest “a large group of the child population” instead of “a large pool of children”

Line 38-40: Suggest “Larger than expected poor treatment outcomes and longer recovery time ~~become more than expected~~ in this model of care compared to inpatient care~~, which~~ might be related to poor implementation and quality of the service”

Line 42-44: Suggest “Assessing the program’s performance, key programmatic variables and clinical outcomes ~~will be identified that are~~ is pivotal in bringing strategies to end childhood death and associated long-term sequalae due to SAM.”

Line 90: suggest the word “address” instead of “battle”

Line 91: suggest the wording “morbid and mortal” rather than “lethal”

Lines 91-93: suggest “The program ~~was~~ launched nationally in 2007 ~~in the country~~, with HEWs employed to support ~~aiming to manage~~ children with SAM and without medical complications by administering medicines and therapeutic food ~~by HEWs”.~~

Line 106: delete “Besides”

Line 108: Delete “Moreover”

Line 124: delete “for the qualitative part”

Line 138-139: suggest “Availability and acceptability dimensions from the access framework and the compliance dimension from the implementation fidelity framework will be evaluated.”

Line 198: suggest “examined by a” is replaced with “examined using a”

Line 207: is “filed” a typo of “field”?

Line 219: suggest “A two-day training programme” and delete “that lasts two days”

Line 241: suggest “data cleaning will be completed” instead of “data cleaning will be done”

Line 249: typo “similarly”

Line 287: replace “won’t” with “will not”

Line 291: describe in more detail that compensation will relate to travel and/or other costs related to taking part in the FGD

Line 292: typo “Mis-classified/diagnosed” (only one ‘s’)
